# Evaluation of ocular biometry in the Japanese population using a multicenter approach: Prospective observational study

**DOI:** 10.1371/journal.pone.0271814

**Published:** 2022-07-27

**Authors:** Takushi Kawamorita, Hiroshi Uozato, Tetsuro Oshika, Kazuno Negishi, Takashi Fujikado, Akira Murakami, Kazutaka Kamiya, Naoyuki Maeda, Yuta Ueno, Kazuhiro Onuma, Masakazu Hirota, Rie Hoshikawa, Sachiko Masui, Masahiro Yamaguchi, Toshifumi Mihashi

**Affiliations:** 1 Department of Orthoptics and Visual Science, Kitasato University School of Allied Health Sciences, Kanagawa, Japan; 2 Tokyo Optometric College, Tokyo, Japan; 3 Faculty of Medicine, Department of Ophthalmology, University of Tsukuba, Ibaraki, Japan; 4 Department of Ophthalmology, Keio University School of Medicine, Tokyo, Japan; 5 Department of Applied Visual Science, Osaka University Graduate School of Medicine, Osaka, Japan; 6 Department of Ophthalmology, Juntendo University Graduate School of Medicine, Tokyo, Japan; 7 Department of Ophthalmology, Osaka University Graduate School of Medicine, Osaka, Japan; 8 Center for Frontier Medical Engineering, Chiba University, Chiba, Japan; 9 Department of Orthoptics, Teikyo University, Tokyo, Japan; National Eye Institute, UNITED STATES

## Abstract

This prospective observational study aimed to evaluate the ocular biometry of Japanese people through a multicenter approach. The uncorrected and corrected distance visual acuity (UDVA and CDVA, respectively) in the log minimum angle of resolution (logMAR), subjective and objective spherical equivalent values (SE) of ocular refraction, anterior and posterior corneal curvature (ACC and PCC, respectively), anterior and posterior corneal asphericity (ACA and PCA, respectively), central corneal thickness (CCT), anterior chamber depth (ACD), and ocular axial length (AL) were measured in the eyes of 250 participants (mean age = 46.5 ± 18.0 years, range: 20–90 years) across five institutions in Japan. The mean UDVA, CDVA, subjective SE, objective SE, ACC, PCC, ACA, PCA, CCT, ACD, and AL were 0.68, −0.08, −2.42 D, −2.66 D, 7.77 mm, 6.33 mm, −0.31, −0.39, 0.55 mm, 2.92 mm, and 24.78 mm, respectively. Age-related changes and sex-based differences were noted in the visual acuity, refraction, corneal shape, ACD, and AL. Our results serve as basis for future studies aiming to develop refractive correction methods and various vision-related fields.

## Introduction

Gullstrand’s eye model, a representative eye model created with reference to anatomical data, plays an important role in the optimal design of eyeglasses, contact lenses, intraocular lenses, and lasers for vision correction. This model is a useful model that can still be used today, however it does not include information such as aspheric surfaces and subjective visual performance. Although many historical eye models have been reported [1,2], more accurate models have been developed owing to recent improvements in the measurement and evaluation techniques of the shape of the eye. In fact, in addition to Gullstrand’s eye model, there are other models of the eye that consider focal adjustment [3] and age-related changes that reflect biometrics [4] among other parameters. Moreover, Liou and Brennan proposed a new model that is closer to anatomical, biometric, and optical realities [5]. Recently, in Europe (EU), a study called Project Gullstrand—European Project for the Determination of Average Biometric Values of Human Eyes (Gullstrand) was conducted by the European Vision Institute Clinical Research Network and reported by Zocher et al. [6]. Furthermore, the Bigaussian model was reported by Rozema et al. in Europe [7]. Although these reports use highly accurate measurement instruments, it is important to compare the EU model with the Asian model because of possible racial differences.

Refractive errors in Japanese and East Asian eyes are often myopic owing to genetic and environmental factors [8,9]; thus, corrections to the eye model may be required to account for these. In myopic eyes, the anterior chamber depth (ACD), defined as the distance from the cornea to the lens, is deeper than that in emmetropic or hyperopic eyes, and the length of the ocular axial length (AL) is longer [10]. These findings suggest that the standard eye shapes are different from Japanese and East Asian eye shapes. Various studies have been conducted on data on the shape or the refraction of the eye, including the Tajimi and Nagahama studies in Japan [11,12] and the Liwan study in China [13].

In this study, we compared the present study with these previous studies [6,11,13] and our eye model with other famous eye models, such as the Gullstrand [2], Navarro [3], and Liou and Brennan eye models [5].

This multicenter study was conducted at institutions belonging to the Japanese Society of Ophthalmological Optics (JSOO). This study aimed to compare the data from the multicenter study with other studies and eye models, thereby allowing optimization of eyeglasses, contact lenses, intraocular lenses, and laser correction, and to apply this information to various fields.

## Materials and methods

This was a multicenter, prospective observational study and a cross-sectional survey involving the following five institutions: Osaka University, Kitasato University, Keio University, Juntendo University, and the University of Tsukuba. A target of 250 eyes was set, and 250 participants were enrolled between October 18, 2016 and March 31, 2019. The inclusion criteria were as follows: measurement of one healthy eye (left or right eye, randomly determined), subjective and objective spherical equivalent values (SEs) of −10.0 diopter (D) to +10.0 D, absence of corneal or retinal lesions, no ocular disease other than cataracts, and residents of Japan. The exclusion criteria were as follows: presence of lesions, no ocular diseases, no previous ophthalmic surgery, no amblyopia, refractive error greater than ±10.0 D, pseudophakic eyes, no systemic diseases (e.g., diabetes and multiple sclerosis), women who were more than five months pregnant prior to testing, and hard contact lens wearers. Eyes with tessellated fundus was not contained in the exclusion criteria.

This study was approved by the Institutional Review Boards of the five institutions abovementioed (Osaka University protocol code 16523–4, Kitasato University protocol code B16-56, Keio University protocol code 20160142, Juntendo University protocol code 16–283, and the University of Tsukuba protocol code H28-100) and have been performed in accordance with the Declaration of Helsinki. Written informed consent was obtained from all participants.

The uncorrected distance visual acuity (UDVA) and best corrected distance visual acuity (CDVA) in the log minimum angle of resolution (logMAR) unit, subjective manifest refraction (spherical and minus cylindrical powers and axis), and objective refraction (spherical and minus cylindrical powers and axis) were evaluated using the autorefractometer. The AL was evaluated using optical low-coherence reflectometry (IOLMaster 500 or 700, Carl Zeiss Meditec AG, Jen, Germany). The radius of the anterior corneal curvature (ACC) and posterior corneal curvature (PCC), anterior corneal asphericity (ACA) and posterior corneal asphericity (PCA), central corneal thickness (CCT), and ACD expressed as the distance from the posterior surface of the cornea to the front surface of the lens were estimated using the Pentacam HR (Oculus, Wetzlar, Germany) with a rotating Scheimpflug photography. The SE and power vectors, J180 and J45, were calculated from the subjective manifest refraction and objective refraction as follows [14]:

$$SE=Sph+\left(\frac{Cyl}{2}\right)$$

$$J180=\left(-\frac{Cyl}{2}\right)\cos\;2\;\alpha$$

$$J45=\left(-\frac{Cyl}{2}\right)\sin\;2\;\alpha$$ (1)

where Sph is the sphere power, Cyl is the minus cylinder power, and α is the minus cylinder axis.

The total corneal power (TCP) was calculated using the follow formula:

$$TCP=ACP+PCP-\left(\frac{CCT}{n2}\right)\left(\frac{n2-n1}{ACC}\right)\left(\frac{n3-n2}{PCC}\right)$$ (2)

where ACP is the anterior corneal power; PCP is the posterior corneal power; CCT is the central corneal thickness; ACC is the radius of the anterior corneal curvature; PCC is the radius of the posterior corneal curvature; and n1, n2, and n3 are the refractive indices of air (1.000), cornea (1.376), and aqueous humor (1.336), respectively. The PCC/ACC ratio and the AL / ACC ratio were also calculated. These measurements were performed under non-mydriatic and non-cycloplegic conditions.

Statistical analyses were performed using the IBM SPSS Statistics software (version 25.0, SPSS, Inc., Chicago, IL, USA) and Microsoft® Excel® for Office 365 (Microsoft Co., Ltd, Redmond, WA, USA). Descriptive statistics (mean, standard deviation, median, minimum value, maximum value, and percentage) were computed and tests of normality, Shapiro-Wilk test, was performed. A regression analysis of age for each parameter was performed to assess changes associated with age. Regression equations were either linear or curve estimations based on the height of the adjusted coefficient of determination. To know if the amount of data was sufficient, we calculated the OTP using α = 0.05. The OTP takes a value between 0 and 1; the closer the value is to 1, the more likely that the analysis of variance (ANOVA) is based on sufficient data.

Non-parametric analyses using the Mann–Whitney U test (for two groups) and the Kruskal–Wallis test (for independent samples) were performed to evaluate the sex-based differences in each parameter between the age groups. Furthermore, an ANOVA and the Games–Howell post-hoc test for multiple comparisons were performed to compare the parameters between the refractive data groups. A P value of less than 0.05 was considered statistically significant.

In addition, the results of this study were compared with those of previous reports and eye models [2,3,5,6,11,13].

## Results

A total of 250 eyes from 250 participants (mean age = 46.5 ± 18.0 years, range: 20–90) were included in this study (126 women and 124 men with mean ages of 46.74 ± 18.41 and 46.25 ± 17.56 years, respectively, P = 0.905). The descriptive statistics are shown in Table 1. The mean logMAR value for CDVA was −0.08 ± 0.09, indicating good vision. The mean logMAR value for UDVA was 0.68 ± 0.53, indicating a high degree of variability and a tendency for many participants to have insufficient vision.

**Table 1:** Descriptive statistics (n = 250).

Parameter	Mean	SD	Median	Min	Max
Age, years	46.50	17.96	45.00	20.00	90.00
UDVA, logMAR	0.68	0.53	0.70	−0.30	1.70
CDVA, logMAR	−0.08	0.09	−0.08	−0.30	0.30
Subjective Sph, D	−2.08	2.91	−1.88	−8.75	7.00
Subjective Cyl, D	−0.68	0.73	−0.50	−3.50	0.00
Subjective SE, D	−2.42	2.89	−2.25	−8.88	6.63
Subjective J180	−0.01	0.44	0.00	-1.72	1.25
Subjective J45	0.02	0.22	0.00	-0.87	1.15
Objective Sph, D	−2.26	3.08	−2.00	−9.50	7.25
Objective Cyl, D	−0.80	0.67	−0.63	−3.75	0.50
Objective SE, D	−2.66	3.05	−2.38	−9.75	6.88
Objective J180	0.02	0.47	0.04	−1.82	1.25
Objective J45	−0.01	0.23	0.00	−0.94	1.15
AL, mm	24.78	1.46	24.78	21.12	28.34
ACD, mm	2.92	0.41	2.94	1.82	3.95
ACC, mm	7.77	0.27	7.77	7.16	8.49
PCC, mm	6.33	0.27	6.33	5.63	7.09
CCT, mm	0.55	0.03	0.55	0.46	0.64
ACA	−0.31	0.13	−0.30	−0.91	0.00
PCA	−0.39	0.18	−0.39	−0.91	0.13
ACP, D	48.46	1.67	48.41	44.29	52.51
PCP, D	−6.33	0.27	−6.32	−7.11	−5.64
TCP, D	42.26	1.45	42.18	38.64	45.58

SD, standard deviation; Min, minimum; Max, maximum.

Owing to the variation in the normality of the various eye shape parameters, a non-parametric test was used. The mean and distribution results of the subjective and objective SE of ocular refraction revealed mild myopia and astigmatism (Table 1). The spherical power (Sph), cylindrical power (Cyl), SE, power vector J180, and power vector J45 for differences between the subjective and objective measurements were 0.180 ± 0.463 D, 0.122 ± 0.329 D, 0.241 ± 0.469 D, −0.033 ± 0.222, and 0.025 ± 0.154, respectively (Table 1). The PCC / ACC and AL/ACC ratios were 0.82 ± 0.02 and 3.19 ± 0.17, respectively. In the normality analyses, the hypothesis "This variable is normally distributed" was rejected for age, UDVA, CDVA, and all subjective and objective refractive parameters (P < 0.05), while AL, ACD, ACC, PCC, CCT, PCA, PCC/ACC ratio, and AL/ACC ratio were accepted (P > 0.05).

A comparison between these results and results from the previous reports is presented in Table 2. Compared to the Gullstrand’s model eye [2], the posterior surface of the cornea was 0.5 mm steeper, and the cornea was 0.05 mm thicker. In the Liou and Brennan’s model eye [5], the ACA and PCA were −0.18 and −0.60, respectively, while in this study, the ACA and PCA were −0.30 and −0.39, respectively; the corneal radius of curvature and thickness were almost the same. Compared to that in the Nagahama study [11], the ACC was 0.10 mm flatter and the ACD was 0.24 mm shallower. Compared to that in the German study [6], the ACC and PCC were 0.05 mm and 0.14 mm steeper, respectively and the ACD was 0.11 mm deeper. In addition, the AL in the present study was longer than that of any ocular model [2,3,5] and ALs reported in the previous studies [6,11,13].

**Table 2:** Comparison of the eye shape results of this study and other eye models and previous studies [2,3,5,6,11,13].

Parameter	Present study	Gullstrand’s model	Navarro’s model	Liou and Brennan’s model^a^	Nagahama study	Liwan eye study	German study^b^
Age, years (Min to Max)	46.5±18.0 (20–90)	n/a	n/a	n/a	57.6±12.4 (34–80)	64.4±9.6 (n/a)	43, 42^d^ (21–69)
ACC, mm	7.77±0.27	7.70	7.72	7.77	7.67±0.25	7.69^b^^,^ ^c^	7.82±0.26
PCC, mm	6.33±0.27	6.80	6.50	6.40	n/a	n/a	6.47±0.25
CCT, mm	0.55±0.03	0.50	0.55	0.55	0.54±0.03	n/a	0.55±0.03
ACA	−0.30±0.13	n/a	−0.26	−0.18	n/a	n/a	0.38±0.19^e^
PCA	−0.39±0.18	n/a	0.00	−0.60	n/a	n/a	0.16±0.36^e^
AL, mm	24.78±1.46	24.39	24.00	23.97	24.09±1.37	23.11^b^	23.80±1.05
ACD, mm	2.94±0.41	3.10	3.05	3.16	3.18±0.38	2.67^b^	2.83±0.37

n/a, not applicable; Min, minimum; Max, maximum.

The values in the table are expressed as the mean ± standard deviation, unless otherwise stated.

^a^Merging multiple reports.

^b^Median values are shown (mean and SD data were not available).

^c^Estimated value was calculated from the equivalent refractive index 1.3375.

^d^43 years in women, 42 years in men.

^e^Eccentricity.

**Fig 1:**
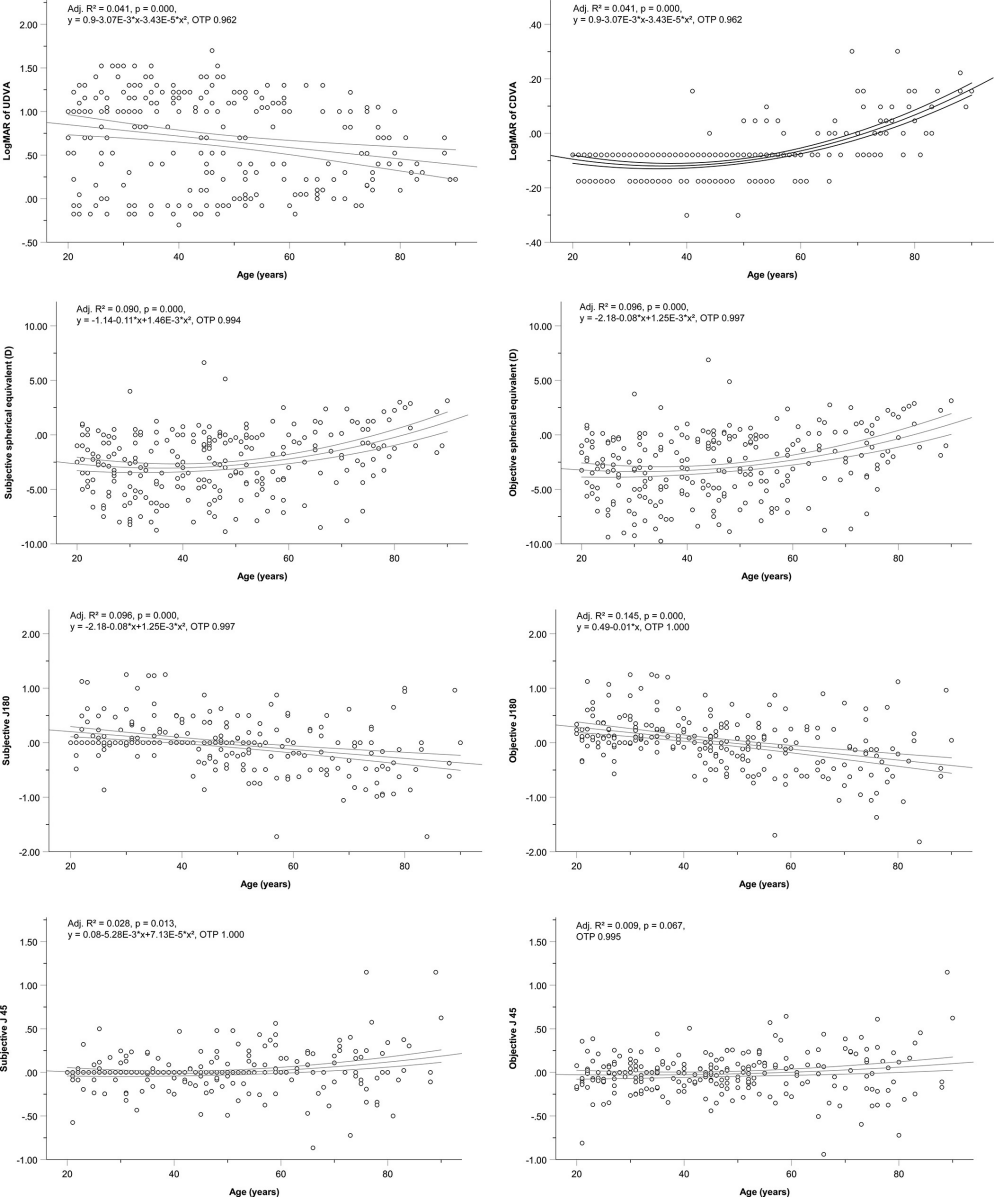
Age-Associated changes in visual acuity and refraction for each study parameter (only significant correlations from Table 3 are shown). **(a)** Log minimum angle of resolution (logMAR) of the uncorrected distance visual acuity (UDVA); **(b)** logMAR of the corrected distance visual acuity (CDVA); **(c)** Subjective spherical equivalent (Subjective SE); **(d)** Objective spherical equivalent (Objective SE); **(e)** Subjective J180; **(f)** Objective J180; **(g)** Subjective J45; **(h)** Objective J45. The three lines represent regression curves and 95% confidence intervals. D, diopter; Adj. R^2^, adjusted R^2^; OTP, observation test power.

**Fig 2:**
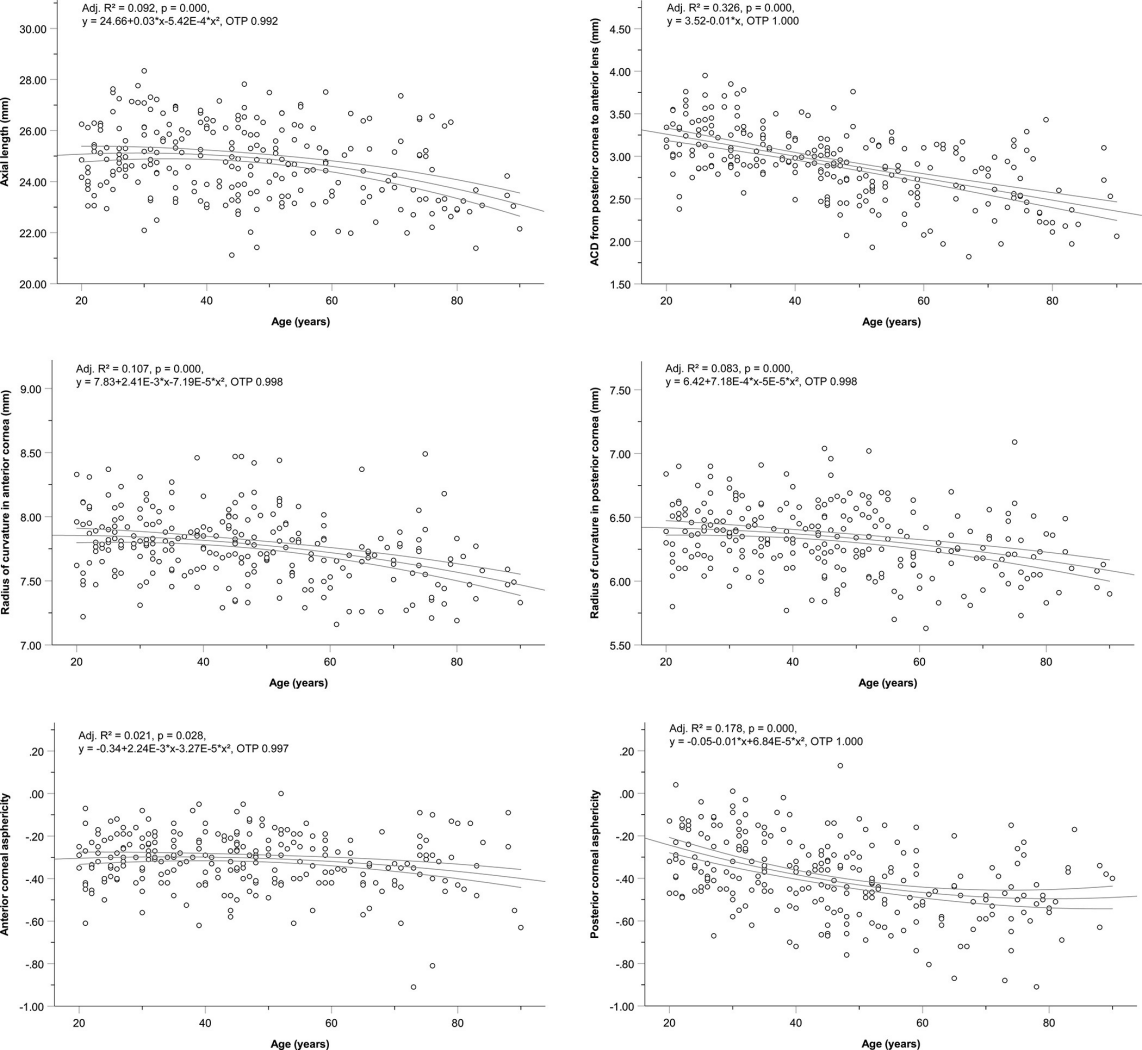
Ocular biometry. Age-associated changes for each study parameter (only significant correlations from Table 3 are shown). **(a)** Axial length (AL); **(b)** Anterior chamber depth (ACD) from the posterior cornea to the anterior lens; **(c)** Radius of curvature of the anterior cornea (ACC); **(d)** Radius of curvature of the posterior cornea (PCC); **(e)** Anterior corneal asphericity (ACA); **(f)** Posterior corneal asphericity (PCA). The three lines represent regression curves and 95% confidence intervals. Adj. R^2^, adjusted R^2^; OTP, observation test power.

**Fig 3:**
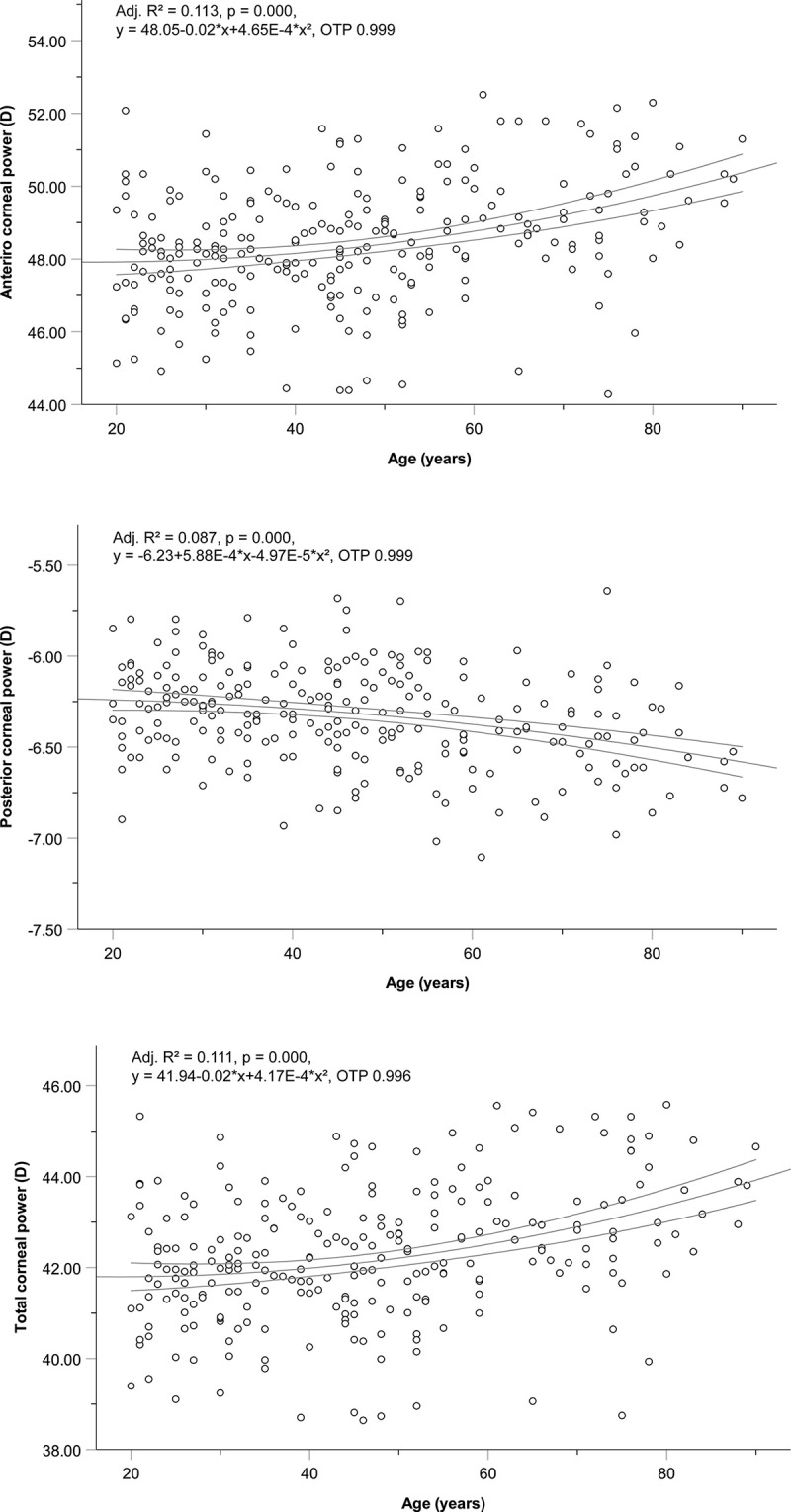
Age-associated changes in the corneal power. **(a)** Anterior corneal power (ACP); **(b)** Posterior corneal power (PCP); **(c)** Total corneal power (TCP). The three lines represent regression curves and 95% confidence intervals. D, diopter; Adj. R^2^, adjusted R^2^; OTP, observation test power.

With respect to age group difference, the visual acuity (UDVA, adjusted R^2^ = 0.041, P < 0.001; CDVA, adjusted R^2^ = 0.437, P < 0.001) and many ocular shape parameters were correlated with age (Figs 1–3, Table 3).

**Table 3:** Comparison between age groups in ocular biometry.

Parameter	Mean±SD per age group						ANOVA p
	20–29 y	30–39 y	40–49 y	50–59 y	60–69 y	over 70 y	
n	50	50	51	42	19	38	-
UDVA, logMAR	0.77±0.53	0.86±0.56	0.65±0.59	0.67±0.49	0.36±0.49	0.50±0.35	0.020
CDVA, logMAR	-0.11±0.05	-0.11±0.04	-0.12±0.07	-0.09±0.07	-0.04±0.11	0.05±0.09	< 0.001
Subjective Sph, D	-2.57±2.33	-3.22±2.98	-2.04±3.02	-2.54±2.67	-1.04±2.63	-0.01±2.66	< 0.001
Subjective Cyl, D	-0.39±0.61	-0.54±0.75	-0.49±0.51	-0.83±0.68	-0.86±0.57	-1.26±0.84	< 0.001
Subjective SE, D	-2.76±2.37	-3.48±2.97	-2.29±3.06	-2.96±2.70	-1.47±2.75	-0.63±2.62	< 0.001
Subjective J180	0.09±0.32	0.21±0.38	0.01±0.32	-0.11±0.47	-0.21±0.39	-0.27±0.6	< 0.001
Subjective J45	-0.01±0.14	0.00±0.14	-0.01±0.15	0.04±0.23	-0.05±0.28	0.11±0.38	0.058
Objective Sph, D	-2.87±2.54	-3.56±3.15	-2.14±3.22	-2.58±2.69	-1.04±2.75	-0.18±2.85	< 0.001
Objective Cyl, D	-0.65±0.55	-0.69±0.70	-0.61±0.45	-0.84±0.64	-1.03±0.62	-1.26±0.87	< 0.001
Objective SE, D	-3.19±2.56	-3.90±3.16	-2.44±3.24	-3.01±2.72	-1.55±2.84	-0.81±2.77	< 0.001
Objective J180	0.18±0.33	0.28±0.37	0.01±0.34	-0.10±0.47	-0.19±0.48	-0.27±0.62	< 0.001
Objective J45	-0.04±0.19	-0.02±0.16	-0.03±0.17	0.04±0.22	-0.09±0.31	0.05±0.36	0.142
AL, mm	25.10±1.24	25.28±1.38	24.74±1.55	24.86±1.32	24.15±1.4	23.97±1.49	< 0.001
ACD, mm	3.22±0.33	3.14±0.27	2.91±0.33	2.71±0.30	2.71±0.44	2.58±0.38	< 0.001
ACC, mm	7.85±0.24	7.82±0.24	7.82±0.27	7.75±0.24	7.61±0.27	7.61±0.27	< 0.001
PCC, mm	6.41±0.23	6.39±0.24	6.37±0.27	6.32±0.27	6.17±0.27	6.21±0.26	< 0.001
CCT, mm	0.54±0.03	0.55±0.03	0.55±0.03	0.55±0.03	0.55±0.02	0.55±0.03	0.593
ACA	-0.30±0.11	-0.29±0.12	-0.30±0.12	-0.30±0.12	-0.37±0.10	-0.36±0.18	0.057
PCA	-0.30±0.15	-0.31±0.17	-0.41±0.17	-0.43±0.15	-0.56±0.16	-0.48±0.17	< 0.001
ACP, D	47.93±1.45	48.11±1.48	48.13±1.66	48.53±1.50	49.46±1.74	49.49±1.72	< 0.001
PCP, D	-6.25±0.23	-6.27±0.24	-6.29±0.27	-6.34±0.27	-6.50±0.29	-6.46±0.26	< 0.001
TCP, D	41.81±1.27	41.96±1.29	41.95±1.44	42.32±1.30	43.09±1.53	43.16±1.52	< 0.001

SD, standard deviation; y, years; ANOVA, analysis of variance.

Concerning visual acuity, the UDVA shifted to the minus side slightly with age (P < 0.001, Fig 1 and Table 3), although there was a large variability. The CDVA shifted to the positive side slightly with age (P < 0.001, Fig 1 and Table 3). For refraction, both subjective SE and objective SE changed to the positive side with age (P < 0.001, Fig 1 and Table 3). Furthermore, the J180 shifted to the minus side with increasing age, i.e., from with-the-rule astigmatism to against-the-rule astigmatism (P < 0.001 for both subjective J180 and objective J180, Fig 1 and Table 3). For J45, there was a trend towards greater variability with age; while no age-related changes were observed in the objective values (P = 0.067, regression analysis; P = 0.142, ANOVA), a positive change toward 135° was noted in the subjective values (P = 0.013, regression analysis; P = 0.058, ANOVA). Furthermore, the observation test power (OTP) was almost equal to 1 (Fig 1).

Ocular biometry shows that the corneal shape is slightly steeper (ACC, adjusted R^2^ = 0.107, P < 0.001; PCC, adjusted R^2^ = 0.083, P < 0.001) and more oblate (ACA, adjusted R^2^ = 0.021, P = 0.028; PCA, adjusted R^2^ = 0.178, P < 0.001) with age (Fig 2). The parameters that did not differ significantly were the CCT (adjusted R^2^ = 0.001, P = 0.244), PCC/ACC ratio (P = 0.447), and AL/ACC ratio (adjusted R^2^ = 0.020, P = 0.074). Furthermore, the OTP in the univariate general linear model was almost equal to 1 (Figs 1 and 2).

Regarding sex-based differences, men had a slightly worse UDVA than women (P = 0.03) due to negative shifts in the subjective and objective SE (P = 0.014, P = 0.020, respectively; Fig 1 and Table 4). Regarding ocular parameters, men had a slightly longer ocular AL (P < 0.001), deeper ACD (P < 0.001), flatter corneal shape (ACC, P < 0.001; PCC, P < 0.001), and different ACA (positive side) than women (P = 0.012; Fig 2 and Table 4). The anterior corneal power (ACP) and total corneal power (TCP) were significantly more refractive, and the posterior corneal power (PCP) was significantly more negative in women than in men (P < 0.001 for ACP, TCP, and PCP; Fig 3 and Table 4). The parameters that did not differ significantly were the CDVA (P = 0.715), subjective and objective cylinder (subjective Cyl, P = 0.201; objective Cyl, P = 0.440), J180 (subjective J180, P = 0.468; objective J180, P = 0.752), J45 (subjective J45, P = 0.601; objective J45, P = 0.890), CCT (P = 0.338), PCA (P = 0.889), and PCC / ACC ratio (P = 0.085) (Table 4).

**Table 4:** Comparison of sex-based differences.

Parameter	Women (n = 126)			Men (n = 124)			Mann–Whitney U test P
	Mean	SD	Median	Mean	SD	Median	
Age, years	46.74	18.41	45.00	46.25	17.56	45.00	0.905
UDVA, logMAR	0.61	0.55	0.70	0.75	0.51	0.82	0.030
CDVA, logMAR	-0.08	0.09	-0.08	-0.08	0.09	-0.08	0.715
Subjective Sph, D	-1.72	2.75	-1.00	-2.45	3.02	-2.75	0.024
Subjective Cyl, D	-0.62	0.70	-0.50	-0.74	0.75	-0.50	0.201
Subjective SE, D	-2.03	2.75	-1.50	-2.82	2.98	-2.75	0.014
Subjective J180	0.00	0.42	0.00	-0.03	0.47	0.00	0.468
Subjective J45	0.01	0.21	0.00	0.02	0.23	0.00	0.601
Objective Sph, D	-1.88	2.94	-1.25	-2.64	3.18	-3.00	0.032
Objective Cyl, D	-0.76	0.62	-0.50	-0.84	0.73	-0.75	0.440
Objective SE, D	-2.26	2.92	-1.81	-3.07	3.14	-3.13	0.020
Objective J180	0.01	0.43	0.06	0.03	0.51	0.00	0.752
Objective J45	0.00	0.23	0.00	-0.01	0.23	0.00	0.890
AL, mm	24.23	1.29	23.98	25.33	1.41	25.30	< 0.001
ACD, mm	2.82	0.38	2.88	3.01	0.41	3.03	< 0.001
ACC, mm	7.68	0.24	7.70	7.86	0.26	7.86	< 0.001
PCC, mm	6.24	0.25	6.23	6.42	0.25	6.41	< 0.001
CCT, mm	0.55	0.03	0.55	0.55	0.03	0.55	0.338
ACA	-0.34	0.14	-0.32	-0.29	0.12	-0.29	0.012
PCA	-0.39	0.17	-0.39	-0.39	0.19	-0.41	0.889
PCC/ACC ratio	0.81	0.02	0.81	0.82	0.02	0.82	0.085
AL/ACC ratio	3.16	0.15	3.15	3.23	0.18	3.21	0.001
ACP, D	49.01	1.55	48.83	47.90	1.59	47.87	< 0.001
PCP, D	-6.42	0.26	-6.43	-6.24	0.24	-6.24	< 0.001
TCP, D	42.72	1.35	42.58	41.78	1.40	41.70	< 0.001

SD, standard deviation.

## Discussion

This multicenter study was to compare ocular data, including age and sex differences, with other studies and ocular models. Results showed that the values of refraction were slightly myopic, and there were age-related changes and sex-based differences in many parameters.

The results of the descriptive statistics regarding refraction showed the low myopia, which could be related to the higher myopia rate among East Asians. Morgan et al. [8] showed that myopia was common among East Asians and reported that 80–90% of middle school graduates were affected. It has also been reported that the curvature of the corneal radius flattens with increasing myopia [15]. Compared to the other model eyes [2,3,5,6,11,13], in this study the anterior surface of the cornea was similar, the posterior surface was slightly steeper, and the asphericity was different. This may be due to the unique structure of the Japanese eye, where only the AL of the eye is slightly longer. In fact, this result was similar to the result of the large-scale Nagahama study conducted by Nakao et al. [11]. The results of the present study showed that the AL was about 0.7 mm longer than that in the Nagahama Study [11], about 1.7 mm longer than that in the Liwan Eye study [13], and about 1.0 mm more myopic than that in the above-mentioned German study [6]; however, the difference in Nagahama study and Liwan eye study may be because our study also included people in their 20s. The shallower ACD of our model, as compared to that of some previous models [1–3,5,11], could be due to the higher average age of the participants and the inclusion of older individuals.

Regarding age-related changes, previous reports have shown that refractive values become hyperopic with increasing age [16]. In addition, the corneal radius of curvature becomes slightly steeper and the asphericity changes slightly [4]. Dubbelman et al. [17] showed that the corneal radius of curvature does not change with age in either the anterior or posterior surfaces, while the asphericity changes slightly. The AL of the eye is slightly reduced [18], and the lens are reported to experience steepening and have a decreased refractive index [19]. In this study, the refraction values became more hyperopic, the corneal radius of curvature became steeper, and the ocular AL became shorter with age, each with statistically significant correlations. Therefore, these age-related changes were similar to those in Nagahara’s study for refraction [11] and in Navarro’s study for the corneal radius of curvature [4]. As for the age-related differences in corneal shape obtained in the present results, they may be due to differences in refraction rather than age-related changes. The decrease in the ACD with increasing age appears to be mainly due to an increase in the lens thickness, which was in line with the findings of a previous report [20]. Although the age-related changes in the eye shape were similar to those previously reported [4,11,20], the average height difference between 20-year-olds was about 10 cm higher than that between 80-year-olds [21], which may have affected the difference in the ocular AL. Zocher et al. [6] reported that there is a relationship between height and ocular AL; the regression equation is 0.0393 mm longer per 1 cm of the height.

Regarding sex-based differences, Roters et al. [22] reported that women have a slightly shorter ocular AL and ACD than men. Dubbelman et al. [17] reported that the anterior and posterior corneal surfaces of men were flatter than those of women. For all other parameters, including asphericity and trends with age, no sex-based differences have been observed. The results of the present study showed sex-based differences in the SE, corneal shape, ocular AL, and ACD. These differences between men and women could be related to a correlation between height and ocular shape [23,24]. A comparison of the results between the present study and those obtained by Zocher et al. [6] regarding age- and sex-based differences showed that the results of this study were on the myopic side for all ages. The male eye showed more myopic refraction and an elongated myopic eye shape than the female eye.

Thus, race, age, and sex differences should be considered when using an eye model to optimize refractive correction. The most commonly used eye models include the Gullstrand’s eye model and the LeGrand’s precision eye model, which are spherical proximal axis models [1]. The Navarro’s eye model is an aspheric model that can consider the effects of off-axis aberration and more closely resembles the shape of the human eye [3]. The Liou and Brennan eye model takes into account the distribution of the lens’ refractive index [5]. The Arizona eye model by Schwiegerling can control the accommodation level with longitudinal chromatic aberration of the eye and longitudinal spherical aberration [25]. Furthermore, Atchison and Thibos have reviewed, in detail, the eye models reported in the past [26]. As biometric techniques develop, new ocular models are emerging, and the role of these models will become increasingly important in elucidating the functional role of ocular structures and in developing new refractive correction methods. Eye models based on biometric data should also be actively applied to the development of corrective methods (which are applicable to the majority of cases) and to the selection of appropriate corrective methods that are customizable, in order to meet the needs of individual patients and address the individual differences in refraction.

A possible limitation of this study was the inclusion of some variability due to differences in the autorefractometers used at the five different institutions. However, the trend of the objective refractive data was similar to that of the subjective data, and we believe that the study conclusion would remain unchanged. In addition, the results provided in this study are from a sample size of 250, which is not sufficient to reflect the whole of Japan. However, we believe that the reliability of the results can be ensured, because the measurements were conducted by specialists using instruments with high accuracy and reproducibility as prospective observational study, and the OTP was close to 1. Further studies are needed that address the limitations. The study findings on the model should be interpreted as being reflective of the characteristics of the Japanese eye rather than of the Japanese population. In addition, it would be desirable to examine the statistical differences between the present model eyes and the model eyes reported previously; however, due to the unavailability of raw data, only simple comparisons of the means and medians were made rather than of the statistical tests of differences. Due to racial, regional, and chronological biases, caution should be exercised in interpreting the data, and it is important to conduct future studies to compare the data statistically.

## Conclusion

This study obtained data on the eye shape and visual acuity of Japanese people through a multicenter approach, although the limitations of the study described above must be considered and interpreted with caution. The results can be used to optimize standard optical designs and laser treatments, such as new eyeglasses, contact lenses, and intraocular lenses, for the Japanese and East Asian population. The data can be applied to other fields such as fields related to vision, electronic displays, and lighting, and to provide comparative data when epidemiological studies are conducted in the future. In the future, it is important to collect data from a larger sample size and to also investigate data outside the scope of this study, including eyes with intense myopia and diseases, to compare with the data from other countries.

## Supporting information

S1 File (XLSX)
